# Validation of the Student Athletes’ Motivation Toward Sports and Academics Questionnaire (SAMSAQ) for Korean College Student-Athletes: An Application of Exploratory Structural Equation Modeling

**DOI:** 10.3389/fpsyg.2022.853236

**Published:** 2022-04-21

**Authors:** Youngjik Lee, Jason Immekus, Dayoun Lim, Mary Hums, Chris Greenwell, Adam Cocco, Minuk Kang

**Affiliations:** ^1^Department of Physical Education, Kookmin University, Seoul, South Korea; ^2^Department of Educational Leadership, Evaluation and Organizational Development, University of Louisville, Louisville, KY, United States; ^3^Department of Sports and Leisure Studies, Far East University, Eumseong-gun, South Korea; ^4^Department of Health & Sport Sciences, University of Louisville, Louisville, KY, United States

**Keywords:** college student-athletes, motivations, validation, ESEM, international psychology

## Abstract

The purpose of this study was to validate the Korean version of the Student-Athletes’ Motivation toward Sports and Academics Questionnaire (SAMSAQ) using exploratory structural equation modeling (ESEM). A total of 412 (men 77%; women 23%) South Korean collegiate student-athletes competing in 27 types of sports from 13 different public and private universities across South Korea were analyzed for this study. ESEM statistical approach was employed to examine the psychometric properties of SAMSAQ-KR. To assess content validity, the SAMSAQ-KR was inspected by a panel of content subject experts. The Athletic Identity Measurement Scale was used to obtain convergent validity. The results of this study illustrated that the SAMSAQ-KR appears to be a robust and reliable instrument.

## Introduction

College student-athletes typically find it difficult to balance their responsibilities between undertaking higher education classes and performing at a competitive level in their respective sports. In general, college student-athletes spend around 20–30 h on the activities that are related to their sport (e.g., training, competitive matches, strength and conditioning, and so on) to maintain their level of competitiveness (Guidotti et al., 2013). This dedication toward athletic performance may hinder the academic achievement of college student-athletes. In addition, it typically affects the student-athletes’ life negatively, which include sustaining injuries, limited social interactions, and limited post-athletic career opportunities (e.g., struggling to find a job outside of sports) (Lopes Dos Santos et al., 2020). Thus, there is a growing concern and interest across various countries regarding the educational development of the dual career of college student-athletes (Gaston-Gayles, 2005; Lupo et al., 2015; Quinaud et al., 2020). For example, many European countries (e.g., Belgium, Germany) provide academic services (e.g., tutoring and time management workshops) to support their college student-athletes’ academic achievement (Aquilina and Henry, 2013). In the United States (US), the National Collegiate Athletic Association (NCAA) has started providing numerous forms of academic support, such as appointing academic advisors and tutors and implementing study hall programs, to engage student-athletes in their academic roles (Huml et al., 2014). Additionally, the NCAA has also implemented many legislative acts to enhance their student-athletes’ academic development (e.g., limiting in-season practice to 20 h per week) (Huml, 2018).

The lack of focus on education for college student-athletes has become one of the major problems in South Korean sports society as well. In South Korean school sports culture, there is a great emphasis on student-athletes’ athletic achievement (Lee and Yu, 2021). The roots of this athletic culture can be found in South Korea’s history. For example, South Korea has historically used sports to bolster the country’s image, placing tremendous pressure on their athletes to achieve Olympic glory (Lee, 2006; Lim and Huh, 2009; Heo, 2011). Due to this culture, South Korean student-athletes are required to devote most of their time to training for their sport. In other words, they are required to sacrifice proper academic opportunities for athletic achievement (Lee and Yu, 2021). In addition, the emphasis on athletic performance over academic achievement for student-athletes in South Korea has perpetuated a culture where student-athletes can openly neglect their academic responsibilities. For example, under the South Korean college sports system, high school student-athletes are able to become college student-athletes without proper academic preparation and credentials (e.g., high school grade point average (GPA) and college entrance test), as South Korean colleges only consider athletic achievements for admissions (Kim, 2011).

Although South Korean student-athletes devote considerable time and energy to their sports, only a handful of South Korean student-athletes actually go on to become professional athletes due to unpredictable variables, such as injuries and family circumstances (Ham, 2003; Huml et al., 2014; Kim et al., 2014). Furthermore, the competitive life span of a South Korean professional athlete is short, with the average age of retired professional athletes at only 23.8 years (Yoo, 2016). In other words, most South Korean college student-athletes will need to integrate back into society as a typical member of South Korean society early in their adulthood without the title of athlete that they lived with their whole life (Otto et al., 2019). However, because these individuals spend more time developing athletic skills than developing academic skills, upon retirement, these South Korean student-athletes struggle to integrate successfully back into society (Lee and Kwon, 2013).

To improve this existing situation that many student-athletes are facing, the Korea University Sports Federation (KUSF) was established as a governing and advisory body for college sports in South Korea in 2010. A total of 110 public and private institutions across the nation are currently members of the KUSF, which is approximately 25% of all the South Korean institutions. The KUSF operates and manages the U-league that offers a total of six sports, including baseball, basketball, ice hockey, soccer, soft tennis, and volleyball. In 2017, in an attempt to mitigate the gap between college student-athletes’ athletic and academic accomplishments, the KUSF implemented a minimum GPA requirement for their member institutions. Based on the requirement, student-athletes who earned a GPA of less than 2.0 in the previous semester were not eligible to participate in the next semester’s U-league.

### Student-Athletes’ Motivation Toward Sports and Academics

It is important to monitor student-athletes’ motivation toward academics and athletics, since motivation can be viewed as being central to their participation in the respective activities (Pestana et al., 2018). To examine the academic and athletic motivation of the college student-athletes in the US, Gaston-Gayles developed and validated the Student-Athletes’ Motivation toward Sports and Academics Questionnaire (SAMSAQ), a 30-item scale to examine college student-athletes’ motivation toward sports and academics (Gaston-Gayles, 2005). The instrument was developed within the framework of the expectancy-value theory (Weiner, 1986), the self-efficacy theory (Bandura, 1977), and the attribution theory (Weiner, 1986), which posits that a students’ motivation to perform their academic and athletic tasks. The multidimensional instrument includes three subscales, student-athletic motivation (SAM; eight items), career athletic motivation (CAM; five items), and academic motivation (AM; 16 items), with responses recorded on a six-point Likert scale (1 = Very strongly disagree to 6 = Very strongly agree). The mean score of all the items included in the respective subscales provides the final score for the subscale, and the SAMSAQ’s factor analytic results have supported the scale’s theoretical three-factor structure. To determine internal validity in particular, Gaston-Gayles conducted exploratory factor analysis (EFA) with a sample size of 153 (Gaston-Gayles, 2005). Initially, a total of 30 items were considered for the analysis, but three items were eliminated due to low factor loadings, low item-to-total correlations, and low reliability.

The SAMSAQ has been validated in different contexts since college student-athletes’ imbalance between academics and athletics is one of the growing concerns of international sports societies. Societal contexts where the SAMSAQ has been validated include Italy (Guidotti et al., 2013), the United Arab Emirates (Fortes et al., 2010), Portugal (Quinaud et al., 2020), and South Korea (Park et al., 2015). Previous reports of the inconsistent factor structures of the SAMSAQ suggest that the instrument may be sensitive to socio-cultural contexts (Corrado et al., 2012; Guidotti et al., 2013; Lupo et al., 2015). The validation of the scale in South Korea allowed for a better understanding of the South Korean college student-athletes’ motivation toward academics and athletics. However, there remain two key issues in the 2015 version that need to be addressed. First, a relatively small number of participants from a single sport were used for the analysis. Second, confirmatory factor analysis (CFA) was employed as the main analysis for the study. However, due to low factor loadings, multiple items were deleted from the original instrument.

It is essential to utilize both EFA and CFA when identifying a new factor structure for a new version of the SAMSAQ, as it takes into consideration the socio-cultural contexts in which the study was conducted. Therefore, most of the previous SAMSAQ validation studies employed both EFA and CFA for their analysis (Fortes et al., 2010; Guidotti et al., 2013; Lupo et al., 2015; Quinaud et al., 2020). However, traditional factor analyses (i.e., CFA and EFA) also have their own shortcomings.

### Exploratory Structural Equation Modeling

The factor analytic procedures provide a powerful, flexible approach to examine the internal structure of the SAMSAQ (Brown, 2015). In general, EFA and CFA are the most popular approaches to investigate the factor structure of psychological instruments. EFA is a data-driven approach to determining the number of empirical factors underlying a set of scale items, while CFA represents a model-based approach to testing an instrument’s factor structure based on *a priori* information to specifying the relationship between observed (e.g., items) and latent (e.g., motivation) variables. For instance, even though an EFA model may be empirically supported, it could still report unacceptable model–data fit with CFA. In addition, CFA is typically associated with problems related to the goodness of fit, measurement invariance across groups, differential item functioning, and differentiation of factors (Marsh et al., 2009). CFA’s overly restrictive independent cluster model (ICM), requiring items to load on only one factor and constraining non-target factor loadings to zero, typically results in an unacceptable model–data fit (Marsh et al., 2011). Furthermore, requiring non-target loadings to be zero can result in higher than expected factor correlations (Asparouhov and Muthén, 2009; Marsh et al., 2010). Thus, these problems and issues have led researchers to question the appropriateness of ICM-CFA models in applied research, including psychological instruments.

Exploratory structural equation modeling is a single-procedure factor analytic approach that combines the features and advantages of EFA and CFA to test an instrument’s multidimensionality (Asparouhov and Muthén, 2009). Specifically, ESEM determines the number of factors according to factor interpretability, model–data fit statistics (e.g., comparative fit index (CFI)), and inspection of model parameters (e.g., factor loadings). In terms of a confirmatory approach, ESEM utilizes a target rotation where target loadings are unconstrained factor loadings, whereas non-target loadings are cross-loadings specified close to zero (Marsh et al., 2014). In summary, ESEM yields an interpretable factor analysis that provides a model-based approach, capturing key components of EFA by allowing cross-loadings that fully identify where the items fall, as well as the measures of the model–data fit like CFA (Morin et al., 2020). Therefore, ESEM can be viewed as a robust and flexible model-based factor analytic approach to examine a scale’s multidimensional structure with its exploratory and confirmatory approaches (Asparouhov and Muthén, 2009; Marsh et al., 2010; Morin et al., 2020).

Beyond testing an instrument’s internal structure, ESEM also allows researchers to examine the group differences in the underlying latent factor means and differential item functioning (DIF) detection by using an ESEM-based factor within a multiple-indicator multiple-cause approach (MIMIC) modeling (Jöreskog and Goldberger, 1975; Muthén, 1989; Holland and Wainer, 1993; Marsh et al., 2014). In terms of DIF, it is identified as the item’s statistical properties (e.g., difficulty and discrimination) that differ between the groups (e.g., demographics) based on equal standing on the latent trait (e.g., student-athletes’ motivations). Unlike multiple-group CFA, the MIMIC model investigates the relationship between the background of participants (e.g., gender) and the factors of ESEM and the value of DIF among item intercepts (Marsh et al., 2014). Within the sports and physical education research, the MIMIC model may be suitable for the analyses of multiple groups to test the measurement invariance, as well as using typically small sample sizes from applied research (Vandenberg and Lance, 2000).

**FIGURE 1:**
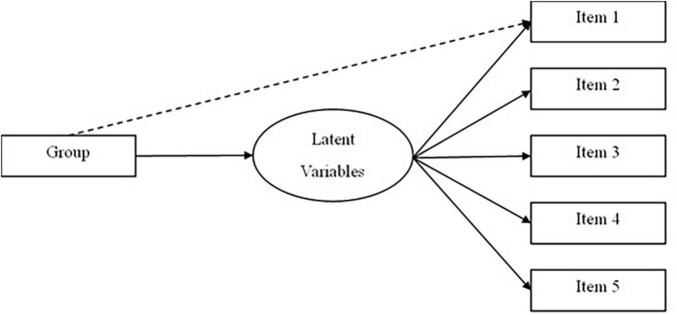
Hypothetical multiple-indicator multiple-cause approach (MIMIC) model.

Figure 1 shows a hypothetical MIMIC model where the latent factor is regressed onto an observed predictor identified by the solid lines, whereas the dotted line indicates the direct effect of the observed predictor on the indicator (e.g., item). In terms of predictor, it could be a categorical (e.g., gender) or continuous variable (e.g., age), with the regression coefficient representing the direction and strength of its relationship with the latent factor. In Figure 1, the solid line indicates the regression coefficient, which provides differences in the latent mean score if the predictor variable indicates gender. In terms of the dotted line, it illustrates that an item represents DIF. The MIMIC model identifies the detection of intercept differences, followed by predicting equality of factor loadings, factor variances, and co-variances across groups. It is important to note that a test of intercept differences needs to be conducted before comparing factor mean scores of the groups because intercept variance is considered as a premise for inspecting latent mean differences.

### Purpose of the Study

With its advantages, there is a growing interest in using ESEM to examine an instrument’s multidimensionality in various fields such as education and medical research field (Dicke et al., 2018; Karlgren et al., 2020; Pommier et al., 2020; Sancho et al., 2020; Neff et al., 2021). In the sports psychology literature as a whole, however, few studies have utilized ESEM to examine the psychometric properties of the instrument as a model-based analysis. Therefore, the purpose of this study was to validate the Korean version of the SAMSAQ using ESEM analysis. Specifically, this study will demonstrate the use of ESEM to investigate the psychometric properties of SAMSAQ based on Marsh et al.’s (2014) ESEM using the following guidelines: First, based on Gaston-Gayles (2005) three-factor model, both ICM-CFA and ESEM model analyses will be conducted to examine which model provides better model–data fit and investigate the comparability of model parameters such as factor loadings. Second, if both models show unacceptable model–data fit, a series of ESEM models will be analyzed using an exploratory approach, which includes various factors to investigate the relationship between ESEM factors and all items. Among a series of ESEM models, a model that provides the best model–data fit and appropriate factor solution will be selected as a preferred model. Subsequently, based on the acceptable model–data fit and factor structure, the MIMC model analysis will be employed to investigate latent mean differences across the student-athletes’ motivation dimensions and the presence of DIF among SAMSAQ items across gender. After examining the psychometric properties of the Korean version of the SAMSAQ, this study will also examine and provide current South Korean college student-athletes’ motivation toward sports and academics across gender. Furthermore, this study contributes to the international sports psychology research field by providing rigorous guidance on how to conduct international research, including (a) how to collect research data from other countries, (b) how to translate an existing scale to other languages systemically, and (c) how to establish the content validity of the translated scale while considering that other countries have different cultures.

## Materials and Methods

### Participants

A total of 435 participants initially responded to the survey. However, 23 responses were removed due to incomplete data. Thus, the remaining 412 responses (men 77%; women 23%) from South Korean collegiate student-athletes competing in 27 types of sports from 13 different public and private universities across South Korea were analyzed for this study. Team sports (i.e., canoe, field hockey, handball, rowing, soccer, and water polo) student-athletes comprised 19% of the sample, whereas student-athletes from individual sports represented 81% of the sample (i.e., archery, badminton, bobsleigh, bowling, boxing, cycling, danceSport, fencing, golf, gymnastics, ice skating, rifle, ski, swimming, taekwondo, tennis, track and field, triathlon, weightlifting, and wrestling). Gender imbalance in South Korean college student-athletes affected the participant composition of this study as well. In particular, in 2020, there were a total of 13,048 college student-athletes in South Korea and 10,299 (80%) were men, whereas 2,749 (20%) were women (Korean Sport and Olympic Committee, 2020).

### Instrumentation

#### Translation Process

First, two bilingual translators (i.e., two Korean doctoral students who are majoring in a sport-related major enrolled in American universities) translated the instrument from English to Korean. Second, for back translation, two bilingual translators (i.e., two Korean faculty members in a sport-related department at two Korean universities who earned their graduate degrees in the United States) who have not been exposed to the instrument were asked to translate the Korean instrument to English. Third, field testing (e.g., interviews with current South Korean college student-athletes) was conducted to ensure that participants could comprehend the questions. Finally, fine-tuning of the instrument included extensive discussions with all the translators if potential discrepancies or problems were identified during the field testing.

#### Student-Athletes’ Motivation Toward Sports and Academics

The SAMSAQ is a 30-item scale to examine the United States college student-athletes’ motivation toward sports and academics (Gaston-Gayles, 2005). The instrument used a total of three different theories: expectancy-value, self-efficacy, and attribution. The instrument consists of three subscales: student-athletic motivation (e.g., It is important for me to do better than other athletes in my sport), career athletic motivation (e.g., My goal is to make it to the professional level or the Olympics in my sport), and academic motivation (e.g., The content of most of my courses is interesting to me), with responses provided on a six-point rating scale (*1* = *Very strongly disagree* to *6* = *Very strongly agree*). The SAMSAQ has reported acceptable levels of internal consistency (α > 0.80) (Taber, 2018).

#### Athletic Identity Measurement Scale

The purpose of the Athletic Identity Measurement Scale (AIMS) is to assess participants’ athletic identity (Brewer and Cornelius, 2001). AIMS will be used to examine convergent validity with the SAMSAQ. AIMS is a seven-item questionnaire with a seven-point rating scale, (*1* = *Strongly disagree* to *7* = *Strongly agree*). Participants’ athletic identity is measured by a total composite score generated by a sum of the scores for the seven items. Higher AIMS scores indicate stronger athlete identification. AIMS has high internal consistency (α = 0.81–0.93) (Taber, 2018). Systemic translation strategies (Sousa and Rojjanasrirat, 2011) were applied to AIMS as well to achieve equivalence in meaning, which required a range of scale translation.

### Procedures

After receiving approval from the University Institutional Review Board (IRB), the researchers developed online versions of the instrument and delivered the instrument to participants through Qualtrics. To reach out to the South Korean student-athletes, the researchers contacted the schools for approval to administer the survey to the student-athletes and to explain the purpose of the study. Upon receiving approval from the schools, the investigator sent a survey link to the coaches of the South Korean college sports teams. Coaches provided the survey link to student-athletes during their team meetings. Student-athletes who wanted to participate in the survey could access the survey on their own devices (e.g., cell phone, laptop, tablet, and so on). The survey contained a total of three sections, (a) demographics, (b) the SAMSAQ, and (c) the AIMS scale, and took approximately 35 min to complete. Prior to taking the survey, all the participants were informed about the research purpose and the option to discontinue the survey at any time. The main data collection phase occurred between August and December 2019.

### Data Analysis

#### Content Validity

Content-related validity was gathered by a panel of experts comprising a former college student-athlete, a Korean professional athlete, a current college student-athlete, and two Korean faculty members with expertise in student-athlete research reviewing the translated version of the SAMSAQ. The panel of experts was provided a translated version of the SAMSAQ to provide feedback regarding item clarity, relevance, and comprehension. Through the content validity process, Item 10 (I chose/will choose my major because it is something I am interested in as a career) was removed since the South Korean college student-athletes are required to major in only sports-related fields, such as physical education, exercise science, and sport management.

#### Statistical Analysis

Descriptive statistics were used to inspect the students’ average ratings and variability of responses. The factor structure of SAMSAQ-KR was guided by the results of Gaston-Gayles (2005) SAMSAQ-US. First, individual ICM-CFA and ESEM models based on Gaston-Gayles (2005) three-factor model were tested. For the ICM-CFA model, a simple model was used where items were loaded on their assigned motivation factors freely, and non-target loadings were constrained to zero. This involved fixing the factor variances to one (Kline, 2013). Parameter estimation was examined based on robust maximum likelihood (MLR) using Mplus 8.4 (Muthén and Muthén, 2005). MLR can be viewed as a suitable analysis to examine fit indices with non-normal, ordered categorical data and to estimate standard errors. Model–data fit was based on the chi-square (χ^2^), the Comparative Fit Index (CFI), the Root Mean Squared Error of Approximation (RMSEA), and the Standardized Root Mean Square Residual (SRMR) index. Acceptable model–data fit criteria included CFI values greater than 0.95, RMSEA less than 0.06, and SRMR less than 0.08 (Hu and Bentler, 1999). Also, Akaike information criterion (AIC) and Bayesian information criterion (BIC) values were examined, with lower values indicating better model–data fit (Schwarz, 1978; Akaike, 1987). Coefficient omega (ω) was examined for factor analytic based estimate of reliability (McDonald, 1999).

#### Convergent Validity

Convergent validity is established when scores on a measure correlate with scores on another measure evaluating a similar construct (Carlson and Herdman, 2012). In this study, the Pearson product-moment correlation was employed to assess the convergent validity of the SAMSAQ-KR by comparing the correlation scores with the AIMS scale. Specifically, the correlation between the scores of athletic-related motivation factors from SAMSAQ-KR and AIMS was investigated.

## Results

The mean values of items varied considerably with values ranging from 1.89 (Item 15: “It is worth the effort to be an exceptional athlete in my sport”) to 5.12 (Item 23: “I am confident that I can earn a college degree”), whereas standard deviation (SD) ranged from 0.98 (Item 23: “I am confident that I can earn a college degree”) to 1.47 (Item 9: “I have some doubt about my ability to be a star athlete on my team”). Table 1 presents the descriptive statistics of the SAMSAQ-KR data for all the 412 participants.

**TABLE 1:** Mean and standard deviation values for SAMSAQ items (*N* = 412).

Item	Mean	SD	Item	Mean	SD
1	4.53	1.33	17	2.98	1.37
2	5.03	1.15	18	4.11	1.33
3	4.58	1.03	19	4.02	1.34
4	4.70	1.15	20	4.73	1.35
5	4.73	1.21	21	3.41	1.29
6	3.46	1.26	22	4.17	1.33
7	4.36	1.07	23	5.12	0.98
8	4.66	1.17	24	4.73	1.08
9	3.73	1.47	25	4.44	1.27
11	3.73	1.46	26	3.49	1.23
12	4.37	1.04	27	4.80	1.08
13	4.87	1.10	28	4.19	1.10
14	2.31	1.14	29	4.15	1.22
15	1.89	1.03	30	4.42	1.35
16	2.94	1.30			

A test based on Gaston-Gayles’s three-factor ICM-CFA model (Gaston-Gayles, 2005) indicated unacceptable model–data fit, where χ^2^(249) = 1,568.708, *p* < 0.01; RMSEA = 0.116 (90% CI: 0.110–0.112); CFI = 0.64; and SRMR = 0.04. Furthermore, a three-factor ESEM model also reported unacceptable model–data fit, where χ^2^(322) = 748.149, *p* < 0.01; RMSEA = 0.057 (90% CI: 0.051–0.055); CFI = 0.90; and SRMR = 0.162. Consequently, a series of ESEM model analyses were conducted to identify the number of empirical factors. As reported in Table 2, the results supported a five-factor solution, where χ^2^ (271) = 456.787, *p* < 0.01, RMSEA = 0.041 (90% CI: 0.034–0.047), CFI = 0.96, and SRMR = 0.03.

**TABLE 2:** Model–data fit of exploratory structural equation modeling (ESEM) models.

Model	χ^2^	*df*	CFI	RMSEA	(90% CIs)	SRMR
3-Factor	748.149*	322	0.90	0.057	(0.051–0.055)	0.04
4-Factor	558.758*	296	0.94	0.046	(0.040–0.052)	0.03
5-Factor	456.787*	271	0.96	0.041	(0.034–0.047)	0.03

*p < 0.01; df: Degrees of freedom. CIs: Confidence intervals.

The EFA factor loading (see Table 3) results showed that the items predominantly loaded onto a specific factor. Five items (e.g., Item 12 and Item 25) did not load on any factor, and one item indicated substantive cross-loadings on two factors (i.e., Item 9). Also, Item 10 (I chose/will choose my major because it is something I am interested in as a career) was eliminated since South Korean college student-athletes are mandated to major in only sports-related fields. Therefore, a total of 23 items from the original version of SAMSAQ were retained and loaded above 0.40 onto one of the five factors.

**TABLE 3:** Item pattern coefficients for final exploratory structural equation modeling (ESEM) solution.

Factors					

Item	1	2	3	4	5
1	**0.95**	–0.17	–0.02	–0.01	–0.07
2	0.17	**0.47**	–0.00	0.34	0.00
3	0.27	0.01	**0.61**	0.01	0.09
4	**0.43**	0.00	0.23	–0.10	0.06
5	0.09	**0.49**	–0.00	0.25	–0.00
6	–0.02	–0.19	0.15	–0.11	**0.49**
7	0.17	0.01	**0.56**	0.00	–0.04
8	0.10	0.39	0.23	0.06	–0.02
9	–0.16	–0.04	0.04	**0.55**	**0.52**
11	0.15	–0.07	0.05	–0.14	**0.49**
12	0.11	0.05	0.00	–0.07	0.00
13	0.18	**0.52**	0.01	0.29	0.00
14	–0.00	**0.57**	0.17	0.29	0.02
15	0.14	**0.65**	0.01	0.16	0.08
16	–0.10	–0.22	0.17	0.04	**0.69**
17	0.00	–0.33	0.37	–0.15	**0.47**
18	–0.09	0.35	–0.05	0.29	–0.15
19	0.07	–0.07	–0.02	**0.83**	–0.14
20	0.03	0.33	–0.01	**0.52**	–0.00
21	0.29	–0.31	0.03	0.14	**0.48**
22	0.09	0.07	0.03	**0.78**	–0.04
23	**0.41**	0.31	0.12	0.03	0.12
24	0.19	0.39	0.26	0.15	0.05
25	0.05	0.34	0.02	0.07	–0.21
26	0.03	–0.01	0.04	–0.10	**0.64**
27	0.13	**0.57**	–0.01	0.34	0.02
28	0.05	–0.02	**0.65**	0.18	–0.02
29	0.12	0.21	–0.03	–0.18	–0.34
30	0.12	0.06	0.18	–0.16	**0.68**

*Bolded values indicate loadings above 0.40. Factor 1: Academic Achievement Motivation; Factor 2: Athletic Motivation; Factor 3: Learning Outcome Motivation; Factor 4: Career Athletic Motivation; Factor 5: Academic Motivation.*

Based on the results of factor loading and acceptable model–data fit, five factors were investigated with a review of all items significantly loading on each factor. Factor 1 was identified as academic achievement motivation and consisted of three items (ω = 0.70) that indicated participants’ academic performance, such as GPA, grades, and graduation. Factor 2 was labeled athletic motivation and included six items (ω = 0.79) that assessed students’ motivation toward their sport. Factor 3 was labeled learning outcome motivation and comprised three items (ω = 0.77) that dealt with students’ learning experiences through their classes and application of that knowledge and skills outside the school upon their graduation. Factor 4 was labeled as career athletic motivation and consisted of three items (ω = 0.85) that included the items representing the participants’ desire to compete at the professional and Olympic levels. Factor 5 was labeled as academic motivation and included a total of seven items (ω = 0.75) that indicated participants’ motivation toward their academic-related tasks.

Table 4 reports the correlation among the ESEM-based factors that ranged from negligible, –0.02 (e.g., Factor 4 and Factor 5) to moderate, 0.50 (e.g., Factor 2 and Factor 4).

**TABLE 4:** Exploratory structural equation modeling (ESEM) factor correlations.

Factors					

	1	2	3	4	5
1	1				
2	0.24	1			
3	0.43*	0.07	1		
4	0.13	0.50*	0.02	1	
5	0.16	−0.00*	0.05*	−0.02*	1

*p < 0.05. Factor 1: Academic Performance Motivation; Factor 2: Athletic Motivation; Factor 3: Learning Outcome Motivation; Factor 4: Career Athletic Motivation; Factor 5: Academic Motivation.

The results of MIMIC modeling reported that the inclusion of direct effects from gender to the items would not improve the model–data fit. Furthermore, the regression coefficients from gender to each of the SAMSAQ-KR factors were non-significant.

Table 5 reports convergent validity results based on the correlation between SAMSAQ-KR’s athletic-related motivation factor (i.e., athletic motivation and career athletic motivation) scores and AIMS. The results indicated that the correlation between athletic-related motivation factors scores and AIMS was moderate (0.59–0.64).

**TABLE 5:** Descriptive statistics and Pearson product-moment correlations between the athletic motivation and career athletic motivation (SAMSAQ-KR) and Athletic Identity Measurement Scale (AIMS).

Variables	Mean	SD	1	2	3
1. AIMS	4.82	0.88	1.00		
2. AM	4.04	0.45	0.62**	1.00	
3. CAM	4.31	1.17	0.59**	0.64**	1.00

** p < 0.01.

## Discussion and Conclusion

The present study aimed to validate the Korean version of the SAMSAQ using ESEM analysis. Compared to the previous studies, which examined South Korean college student-athletes’ motivation toward one sport with a relatively small group of participants, the current study used a larger sample size (n = 412) from 27 different sports types for the analysis. Additionally, ESEM is seldom used in sports psychology research. This study estimated South Korean college student-athletes’ motivation toward academics and athletics, accounting for gender. To ensure content validity, a panel of experts (i.e., both former and current college student-athletes, a current professional athlete, and Korean faculty members with expertise in student-athlete research) reviewed the translated version of the SAMSAQ. In addition, convergent validity was obtained by comparing correlation scores with the AIMS scale. In conclusion, the SAMSAQ-KR proved to be a robust scale with good psychometric properties.

Overall, South Korean student-athletes recognized the importance of academics. This change may be attributed to the recent effort of the government of South Korea to help their college student-athletes academically. For example, the KUSF was established as a governing body of South Korean college student-athletes. The main aim of the KUSF is to facilitate student-athletes’ academic enhancement. For example, the KUSF recently made and implemented the minimum required GPA for student-athletes, supported many colleges by investing in resources for designing academic advisor programs, and conducted seminars for student-athletes regarding academic importance and future career. With the establishment and academic policy implementation of the KUSF, South Korean college student-athletes have many opportunities to recognize the importance of balancing their athletic and academic activities.

Compared to the previous cross-cultural studies that examined student-athletes’ motivation toward athletics and academics used SAMSAQ, the results of this study demonstrated that the SAMSAQ-KR version was characterized by a different factor structure compared to the versions followed by other countries. For example, most previous studies sustained the three-factor structure by utilizing the CFA (Fortes et al., 2010; Guidotti et al., 2013; Lupo et al., 2015; Quinaud et al., 2021). However, the results of this study illustrated that SAMSAQ-KR is composed of a total of five factors: athletic motivation, academic motivation, career athletic motivation, academic achievement motivation, and learning outcome motivation. The results also confirmed that each country has unique socio-cultural contexts and different relationships between the academic and athletic environments in college sport (Fortes et al., 2010; Lupo et al., 2017; Quinaud et al., 2021).

Among all the factors, academic achievement motivation and learning outcome motivation were conceptually equivalent to academic motivation factor in Gaston-Gayles (2005)’s original version of SAMSAQ, since these new factors are closely related to academic-related motivation, such as earning a high GPA (Item 1) and focusing on class content (Item 28). In particular, in SAMSAQ-KR, the academic achievement motivation factor was affected by the recent academic environments of both South Korean high school and college student-athletes. In 2013, the ministry of education in South Korea made and implemented the new rules to enhance the academic performance of South Korean student-athletes. One of the main rules was the minimum required GPA, which mandates high school student-athletes to meet the required GPA to play their sports. In addition, in 2017 (as mentioned earlier), the KUSF was established to manage and support South Korean college student-athletes’ academic performance. KUSF’s initial new rules included minimum required GPA as well. Thus, South Korean college student-athletes were able to recognize the importance of not only their academic performance but also their academic achievement during their stay in both high school and college.

Similar to the previous studies that investigated the college student-athletes’ motivation by gender (Gaston-Gayles, 2005; Sherry and Zeller, 2014), the present study illustrated that South Korean female college student-athletes showed higher motivation toward all academic-related factors compared to the male student-athletes (see Table 6). This observation might be due to South Korea’s male-dominated sports-related career environment. In 2020, for example, there were a total of 7,512 professional athletes in South Korea and 4,834 (64%) were male athletes, whereas 2,678 (36%) were female athletes (Korean Sport and Olympic Committee, 2020). In addition, in terms of the coaching positions, the number of coaches from all levels of sports, including both non-professional (e.g., school sports) and professional levels, in South Korea was 20,593. Of them, the number of male coaches was 16,938 (82%), whereas the number of female coaches was only 3,665 (18%) (Korean Sport and Olympic Committee, 2020). In other words, male college-graduated athletes have more opportunities to retain their sports career after their graduation in the sports-related field, such as professional athletes and coaches. However, female athletes would need to look for future careers that are not related to sports due to a lack of resources and opportunities for college-graduated female athletes. Thus, female college student-athletes might be more focused on academics rather than athletics to earn career opportunities outside of sports after their graduation.

**TABLE 6:** Mean and standard deviation values for motivation scores by gender.

	Academic Achievement Motivation	Athletic Motivation	Learning Outcome Motivation	Career Motivation	Academic Motivation
Male	4.6 ± 1.0	4.3 ± 1.3	3.9 ± 0.9	4.3 ± 1.2	3.6 ± 1.2
Female	4.9 ± 0.9	3.7 ± 1.3	4.5 ± 0.8	4.0 ± 1.2	4.0 ± 1.1

The findings of this study could provide practical implications for the administrators of South Korean college athletics. For example, as discussed earlier, the KUSF has been trying to support and enhance the academic performance of South Korean college student-athletes. However, there is no suitable scale for examining student-athletes’ academics and athletic motivation. Thus, the administrators of South Korean college athletics will be able to use the SAMSAQ-KR scale to analyze and evaluate South Korean college student-athletes’ motivation toward athletics and academics. This will allow administrators to evaluate the impact of the academic policies on the student-athletes by examining their motivation toward academics and athletics. In addition, the faculty members might also use this scale to develop their class materials. For example, the results of this study illustrated that the participants are highly oriented toward learning outcome motivation, which is related to student-athletes’ learning experiences through their classes and application of the knowledge and skills outside of school premises upon their graduation. Therefore, the faculty members should recognize/understand student-athletes’ desire to acquire practical knowledge and skills through the classes and try to make an effort to develop their classes by reflecting student-athletes’ learning outcome motivation. Furthermore, the present study confirmed that South Korean female college student-athletes lay greater emphasis on academics than their male counterparts due to reduced opportunities in sports-related careers after their graduation. Therefore, a different approach, depending on gender, will be needed at the time of making new academic policies for South Korean college student-athletes.

Despite the contributions, the current study also has some limitations that need to be considered. Even though the number of college student-athletes analyzed in this study met the criteria for psychometric analysis, it would be better if future studies include more student-athletes from other sports for the analysis. In addition, although considering the imbalance in gender representation in South Korea’s college sports, that male student-athletes were 10,001 (79%) out of 12,695, the sample of the current study was comprised of mostly male student-athletes. Thus, further research should be conducted with more female college student-athlete for a better interpretation of SAMSAQ-KR.

## Data Availability Statement

The datasets presented in this article are not readily available because additional studies are undergoing using the dataset. Requests to access the datasets should be directed to YL, youngjik.lee@kookmin.ac.kr.

## Ethics Statement

The studies involving human participants were reviewed and approved by University of Louisville Human Subjects Research Institutional Review Board. Written informed consent for participation was not required for this study in accordance with the national legislation and the institutional requirements.

## Author Contributions

YL, MK, and JI conceptualized the study. YL, JI, CG, and AC analyzed the data. YL and DL drafted the manuscript. MH reviewed and edited the manuscript. All authors were involved in designing the study and procedures, read and agreed to the published version of the manuscript.

## Conflict of Interest

The authors declare that the research was conducted in the absence of any commercial or financial relationships that could be construed as a potential conflict of interest.

## Publisher’s Note

All claims expressed in this article are solely those of the authors and do not necessarily represent those of their affiliated organizations, or those of the publisher, the editors and the reviewers. Any product that may be evaluated in this article, or claim that may be made by its manufacturer, is not guaranteed or endorsed by the publisher.
