# Supplementary material for: Validation of the Student Athletes’ Motivation Toward Sports and Academics Questionnaire (SAMSAQ) for Korean College Student-Athletes: An Application of Exploratory Structural Equation Modeling
Source: Front Psychol. 2022 Apr 21;13:853236. doi: 10.3389/fpsyg.2022.853236 (PMC9069065; doi:10.3389/fpsyg.2022.853236)

# *Supplementary Material*

## Supplementary Figures

**Figure 1.** Hypothetical multiple-indicator multiple-cause approach (MIMIC) model.

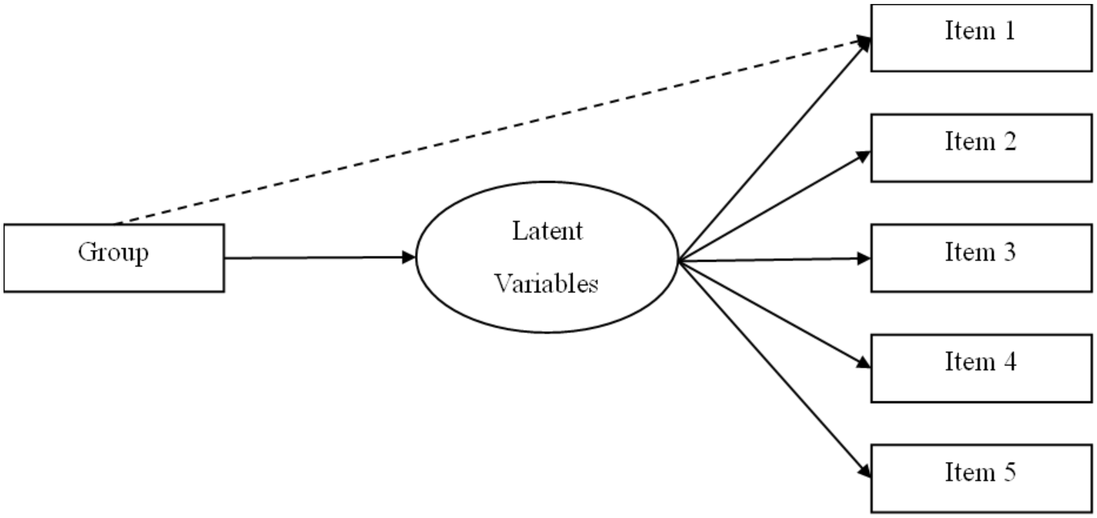

Supplement: Supplementary file 1 [file Image_1.pdf]
